# Sleepless in Solitude—Insomnia Symptoms Severity and Psychopathological Symptoms among University Students during the COVID-19 Pandemic in Poland

**DOI:** 10.3390/ijerph19052551

**Published:** 2022-02-23

**Authors:** Karolina Fila-Witecka, Monika Malecka, Adrianna Senczyszyn, Tomasz Wieczorek, Mieszko Wieckiewicz, Dorota Szczesniak, Patryk Piotrowski, Joanna Rymaszewska

**Affiliations:** 1 Department and Clinic of Psychiatry, Wroclaw Medical University, 50-367 Wroclaw, Poland; k.fila-witecka@umw.edu.pl (K.F.-W.); monika.malecka@student.umw.edu.pl (M.M.); adrianna.senczyszyn@student.umw.edu.pl (A.S.); tomasz.wieczorek@student.umw.edu.pl (T.W.); dorota.szczesniak@umw.edu.pl (D.S.); patryk.piotrowski@umw.edu.pl (P.P.); joanna.rymaszewska@umw.edu.pl (J.R.); 2 Department of Experimental Dentistry, Wroclaw Medical University, 50-425 Wroclaw, Poland

**Keywords:** sleep, insomnia, loneliness, psychopathology, stress, COVID-19 pandemic

## Abstract

Since 25 March 2020, all schools, colleges, and universities in Poland have indefinitely closed and, where possible, have activated distance learning because of the COVID-19 pandemic. Considering that the undergraduate years are usually characterized by a high prevalence of emotional disorders and sleep problems, it can be expected that the current situation may have a remarkable impact on the student population. This study aimed to investigate the occurrence of sleep problems among Polish university students as well as the relationship of insomnia symptoms severity with psychopathological symptoms, posttraumatic stress disorder (PTSD) symptoms, and behavioral factors, such as substance use, changes in the amount of sleep, and the level of physical activity during the COVID-19 pandemic. Data were collected from 1111 Polish university students via an online survey conducted between IV and VI 2020. The survey included demographic variables, the level of psychopathological symptoms (General Health Questionnaire, GHQ-28), insomnia (Insomnia Severity Index, ISI), and symptoms of posttraumatic stress (Impact of Events Scale-Revised, IES-R). The results showed that over half of the studied group of students had some form of sleep disturbances during the period of data collection, with moderate-to-severe insomnia symptoms noted in 21.6%. At the same time, the majority of the sample declared they slept more during the pandemic. A significant positive correlation was observed between the severity of insomnia symptoms and PTSD symptoms, as well as GHQ scores, increased substance use, and decreased physical activity. An additional association between the presence of dreams related to the event and insomnia symptoms as well as GHQ scores has been found. The results suggest that sleep problems may be prevalent among university students during the pandemic. Moreover, although the symptoms of insomnia, as well as the severity of sleep disturbance, significantly correlated with all the investigated variables, the direction of those associations remains to be established.

## 1. Introduction

As Coronavirus Disease 2019 (COVID-19) spreads at an alarming rate, the world has taken drastic measures to contain the spread of the virus. By April 2020, nearly half of the world’s population was under restrictions, with governments encouraging or ordering more than 3.9 billion people to stay at home. According to UNESCO, more than 160 countries implemented nationwide closures, affecting over 87% of students worldwide [1]. In Poland, measures taken to contain the spread of COVID-19 were a nationwide ban on public events, obligation to cover the mouth and nose when outside, self-isolation for symptomatic individuals, and temporary suspension of businesses such as beauty salons. Furthermore, since 25 March 2020 (with a brief break in September), all schools, colleges, and universities have indefinitely closed and, where possible, have activated distance learning as an alternative. Now, almost two years later, most schools still remain in online or hybrid (online and in person where necessary) learning regimes.

In addition to changes in learning, students have lost interaction with colleagues and have been experiencing social isolation, separation from family, grief, fear of losing part-time jobs, and uncertainty about the future as a result of the prediction of global recession due to the COVID-19 pandemic [2]. Considering that undergraduate years are usually characterized by a high incidence of emotional disorders [3,4,5], it can be expected that the current situation may have a remarkable impact on the student population. For instance, a cross-sectional online survey conducted with 44,447 college students from China revealed that the respondents experienced symptoms of anxiety (7.7%) and depression (12.2%) during the COVID-19 pandemic [6]. Another Chinese study also showed that up to 2.7% of quarantined students experienced clinically significant posttraumatic stress disorder (PTSD) symptoms [7]. The European research data on students’ mental health are even more alarming. For instance, a study on a large French sample (1,600,000 respondents) indicated that during the national quarantine, psychopathological symptoms such as suicidal thoughts and high level of perceived stress, depression, and anxiety were present in 11.4%, 24.7%, 16.1%, and 27.5% of the studied population, respectively. It seems that poor mental condition caused by the pandemic may have led the students to consume increased amounts of psychoactive substances and tobacco, as numerous clinical studies [8] and national epidemiological surveys [9] indicated that most substance use disorders were positively significantly correlated with mood and anxiety disorders. Several studies conducted specifically on students have also shown such a correlation [10,11,12].

To date, a vast body of research addressing the pandemic’s impact on sleep quality and changes in sleep habits has emerged [13,14]. Large populational studies, e.g., an international, multicenter survey of 22,330 adults conducted by Morin et al. (2021), reported close to 37% of the studied population reported clinical insomnia symptoms, with over 17% of them meeting the diagnostic criteria of a probable sleep disorder [13]. In another study on 6519 Italians, the reported prevalence rates of insomnia during the pandemic reached over 50% of the entire sample [14]. Importantly, the aforementioned survey was distributed among universities, suggesting a large part of the sample consisted of students. According to Lund et al., sleep quality is poor in over 60% of all college students [15]. While sleep problems are common at the best of times, experiences of isolation, economic and health difficulties, or problems in the relationship with classmates or colleagues, the resulting symptoms of distress, anxiety, and depression may further worsen the existing sleep difficulties. A study by Sara Marelli et al. showed later bedtimes, longer sleep latency, and later wake-up times during the COVID-19 pandemic compared with the prepandemic period [16]. Moreover, it has been shown that, although students generally spend more time in bed, the overall sleep quality worsened during the pandemic, especially among socially isolated university students [17,18]. In addition, during the pandemic, people tend to spend less time outside, and their level of physical activity decreases [19,20], which may have a negative impact on their natural sleep–wake cycle or circadian rhythm. Moreover, as mentioned before, during confinement, people tend to reach for psychoactive substances, which reduce the rapid eye movement (REM) sleep and cause sleep disruptions [21], leading to an increase in the likelihood of PTSD and depression symptoms.

This study aimed to examine the responses of university students to the COVID-19 pandemic and subsequent lockdowns, as well as the resulting isolation and changes in their environment. The study focuses on sleep quality, the presence of psychopathological and PTSD symptoms, and changes caused by the pandemic in behavioral factors, such as alcohol and substance use and physical activity. The purpose of the cross-examination of psychological variables and behavioral changes induced by the pandemic was to establish the current prevalence of sleep problems in the population of students, with the inclusion of factors that are known to contribute to insomnia symptoms. The focus of the study was to examine several variables and their relationships with insomnia symptoms in the studied group during the pandemic: (i) factors with an already established relationship with insomnia symptoms that may or may not have changed during the pandemic (i.e., psychopathological symptoms and previous insomnia symptoms), (ii) factors that are likely to have changed (PTSD symptoms), and (iii) factors that are exclusively associated with the pandemic (behavioral changes, i.e., substance use, changes in sleeping pattern and physical activity).

## 2. Materials and Methods

### 2.1. Study Design

This is a cross-sectional observational study, which used data collected between May and June 2020 using the Computer-Assisted Web Interviewing (CAWI) method. The survey was conducted online, and the form was distributed through social media, university websites, and social media and institutional e-mails among various universities across Poland. The survey was anonymous, and informed consent was obtained from all students participating in the study. Apart from informed consent and sending in a complete set of answers, the participants included in the analysis had to have declared a university student status at the point of data collection. All university students were included, regardless of age and current year of studies. To manage negative emotions related to the study, the respondents were informed that they could contact the psychiatry clinic of the Wroclaw Medical University via e-mail and receive free psychological counseling, following participation, which resulted in a number of participants seeking mental health counseling. The study was conducted according to the guidelines of the Declaration of Helsinki, and the protocol was approved by the Ethics Committee at the Wroclaw Medical University in Poland (no. 309/2020). The study received funding from the Wroclaw Medical University (SUBZ.C230.22.062). The collected data were first analyzed in terms of students’ perspectives on online learning and their adaptation to changes in academic learning and psychological distress [22], as well as in terms of pandemic-related lifestyle changes and their influence on students’ mental health [23].

### 2.2. Participants

The studied sample included 1111 participants who volunteered to take part in the study and submitted a complete set of responses. The majority of participants were female (*n* = 842, 75.79%). The mean age of the studied group was 22.20 ± 2.42 years. The distribution of participants between large and smaller cities was similar, with 531 (47.79) respondents living in small cities with <100 thousand residents and 580 (52.21) living in larger cities with >100 thousand residents. The distribution of singles and people in a relationship was also similar (*n* = 528, 47.52% and *n* = 558, 50.23%, respectively). The majority of the studied participants were unemployed (*n* = 832, 74.89%) and supported by their families (*n* = 929, 83.62%). The demographic characteristics of the study group are described in Table 1.

### 2.3. Measures

The online survey consisted of three sections of questions: (1) sociodemographic information, including gender, age, residence, employment status, income source, and relationship status; (2) lifestyle changes due to the pandemic (3) psychological distress (General Health Questionnaire, GHQ-28), symptoms of posttraumatic stress (Impact of Events Scale Revised, IES-R), and symptoms of insomnia (Insomnia Severity Index, ISI). The internal consistency of the questionnaire evaluating lifestyle changes calculated using Cronbach’s alpha was α = 0.701, while that of the questionnaires evaluating mental health and insomnia symptoms was α = 0.941 (GHQ-28), α = 0.863 (IES-R), and α = 0.863 (ISI). 

The GHQ-28 [24] is a 28-item, self-administered screening tool used to assess short- and long-term changes in mental health. The questionnaire consists of four subscales: somatic symptoms, anxiety and insomnia, social dysfunction, and severe depression. The items are rated on a 4-point Likert scale, ranging from 0 (not at all) to 3 (much more than usual). Typically, higher scores indicate a higher possibility of the presence of psychopathological symptoms. For this study, the cutoff score indicating psychological distress was taken as 24 points.

The IES-R [25] is a self-administered questionnaire consisting of 22 items that are rated on a 5-point Likert scale. The questionnaire assesses, at a subjective level, the presence of symptoms resulting from a traumatic event. The questions are related to three dimensions encompassing PTSD symptoms, namely intrusions, arousal, and avoidance.

The ISI [26] is a brief screening tool to assess sleep problems and the severity of insomnia symptoms. It includes seven items rating sleep onset, sleep maintenance, problem awakening, dissatisfaction with sleep, impairment in quality of life, interference of insomnia with functioning, and distress due to sleep problems. Based on the total scores, insomnia symptoms are graded as follows: absence of insomnia (0–7), subthreshold insomnia (8–14), moderate insomnia (15–21), severe insomnia (22–28) [26].

The questionnaire assessing lifestyle changes during the pandemic was specifically designed for this study and deemed reliable with an internal consistency at Cronbach’s alpha α = 0.701. The questionnaire consisted of 30 items designed to assess subjective changes in everyday life behaviors as well as symptom experiences during the pandemic, i.e., in physical activity, sleeping patterns, and substance use (e.g., “During the pandemic has the average time you spend on sleep/physical activity changed?”). The answers were typically graded as: not applicable, behavior/symptoms increased, behavior/symptoms decreased, no change in behavior/symptoms was noted. The specific wording of the answers was dependent on the questions, e.g., I never drank alcohol, my alcohol consumption decreased, my alcohol consumption increased, my alcohol consumption did not change during the pandemic, or I sleep as much as usual, I sleep more, I sleep less during the pandemic, etc. For both the wording of the questions and the answers, we aimed to design them in a manner that would be precise but still understandable to the population, and some of the terms may be culturally biased. For example, the Polish equivalent of the term tranquilizers was used, which would translate to describe any substance taken with the intent to decrease anxiety, including sleeping aids (i.e., anxiolytics, hypnotics, antipsychotics), as well as over the counter anxiety or sleeping aids and illegal drugs.

### 2.4. Data Analysis

The results obtained for quantitative variables (e.g., IES-R) were presented as mean, standard deviation (SD), median, and interquartile interval, and for categorical variables (e.g., ISI, gender) as count and percentage. The normality of data was assessed using the Shapiro–Wilk test. The difference in demographic variables between the insomnia groups was analyzed using the Kruskal–Wallis, Chi-squared, or Fisher’s exact test, while the difference in the IES-R and GHQ scores between them was analyzed using the Kruskal–Wallis test with post hoc analysis of multiple comparisons with Holm correction. The effect size was described as eta-squared. The relationship between the ISI total score and the IES-R score was assessed using the Spearman correlation coefficient, while the relationship between insomnia and substance use, as well as the physical activity items on the questionnaire, was assessed using the Chi-squared test with a post hoc analysis of multiple comparisons with Holm correction. The relationship between the insomnia groups and the GHQ cutoff point (>24) was assessed using the Chi-squared test with post hoc analysis of multiple comparisons with Holm correction. All analyses were performed in R for Windows, version 4.1 (R Foundation for Statistical Computing, Vienna, Austria), and *p* < 0.05 was set as the significance threshold [27].

## 3. Results

### 3.1. Main Findings

The analysis of the distribution of insomnia symptoms severity across demographic variables, i.e., gender, age, place of residence, relationship status, source of income, and employment, did not reveal any statistically significant differences between the studied groups. The detailed results of the demographic distribution can be found in Table 1.

#### 3.1.1. Relationship between Psychological Distress, PTSD Symptoms, and Insomnia Symptoms Severity

The results indicated a significant relationship between insomnia symptoms severity (ISI) and psychopathological symptoms (GHQ-28). This trend was observed for all GHQ subscales (somatic symptoms, anxiety and insomnia, social dysfunction, severe depression) with moderate-to-large effect sizes. The post hoc analyses revealed significant differences between the studied groups in the somatic symptoms, anxiety and insomnia, and severe depression subscales, as well as in the GHQ total score. The post hoc analyses for the social dysfunction subscale also revealed significant differences between the compared groups, except for the differences between groups with severe and subthreshold insomnia symptoms as well as between the groups experiencing severe and moderate insomnia symptoms, which were insignificant. The results are described in detail in Table 2.

When considering the cutoff point for psychological distress (GHQ-28), which was established at >24, the same relationship between GHQ scores and insomnia symptoms emerges. The highest prevalence of severe insomnia symptoms (*n* = 40, 98%) was observed in the group who scored above the cutoff point compared with the group who scored below the cutoff (<24 group) for psychopathological distress, in which only one person reported severe insomnia symptoms (*n* = 1, 2.4%). Similarly, in the group with moderate insomnia symptoms, 97% scored above the cutoff for psychopathological distress (compared with 2.5% in the <24 group), and in the group with subthreshold insomnia symptoms, 88% scored above the cutoff (compared with 12%). Interestingly, the majority of respondents who reported no insomnia (absence of symptoms) also scored above the cutoff for psychological distress. The post hoc analysis revealed significant differences between all the compared groups, except for the comparison between the groups with subthreshold and severe insomnia symptoms.

#### 3.1.2. Insomnia Symptoms Severity and PTSD Symptoms

A significant positive correlation was found between PTSD symptoms in all IES-R subscales and the severity of insomnia symptoms. The lowest IES-R total scores were found for the group with no insomnia symptoms (mean = 27.61, SD = 17.24, *p* < 0.001) and the highest scores for the group with severe insomnia symptoms (mean = 50.8, SD = 18.74, *p* < 0.001), with a large effect size (0.15944). The post hoc analysis revealed significant differences between all the compared groups, except in the case of the groups who experienced severe and moderate insomnia symptoms, for which the differences were not statistically significant. Similarly, a significant relationship was found between PTSD symptoms and insomnia symptoms severity. An increase of PTSD symptoms was observed with an increase in the severity of insomnia symptoms, and significant post hoc difference, excluding the comparison between moderate and severe insomnia symptoms, was found for the intrusion subscale (*p* < 0.001) and the hyperarousal subscale of IES-R (*p* < 0.001), both with a large effect size (effsize = 0.15749 and 0.23844, respectively). The avoidance subscale of IES-R also demonstrated a relationship with PTSD symptoms; however, the post hoc analysis showed significant differences only between the groups with no insomnia symptoms and those with severe, subthreshold, and moderate insomnia symptoms (*p* < 0.001). Small effect size was noted with this subscale (0.03722). The results are described in detail in Table 3.

In the comparison between IES-R scores, ranging from no PTSD, through probable, subclinical, and severe PTSD symptoms, the majority of the group who presented with severe insomnia symptoms also reported severe PTSD symptoms (*n* = 21, 71%). The lowest insomnia symptoms (absence of symptoms) were found in the group who did not present any PTSD symptoms (*n* = 187, 40%). However, as observed for psychological distress, where the highest percentage with no insomnia symptoms was found in the group that scored above the cutoff for GHQ, the second largest group with no insomnia symptoms were those who experienced severe PTSD symptoms (*n* = 147, 31%). The results are described in detail in Table 4.

A separate investigation was launched into one item of the IES-R relating to reoccurring dreams or nightmares about the event and ISI, as well as GHQ scores. The presence of nightmares demonstrated a weak positive correlation with all of the GHQ subscales (total score, somatic symptoms, anxiety and insomnia, social dysfunction, severe depression), as well as the ISI total score at *p* < 0.0001 (r = 0.307, r = 0.292, r = 0.287, r = 0.218, r = 0.292, r = 0.302, respectively). Significant differences were also found for the item and ISI categories (*p* < 0.0001), with post hoc analyses pointing to significant differences between the absence of insomnia symptoms (mean = 0.62; SD = 1.11) and all of the other categories (subthreshold, moderate, severe insomnia symptoms, mean = 1.16; SD = 1.39 and mean = 1.61; SD = 1.55, mean = 1.76; SD = 1.68, respectively) as well as between subthreshold and moderate insomnia symptoms.

#### 3.1.3. Insomnia Symptoms Severity and Substance Use

Alcohol|The majority of the respondents (43%, *n* = 479) reported decreased alcohol consumption compared with the prepandemic period, while 26% (*n* = 296) reported that they had made no change in alcohol consumption and 18% (*n* = 198) declared increased alcohol consumption compared with the prepandemic period. Only 12% of the studied group reported that they never used alcohol. The highest percentage of severe and moderate insomnia symptoms was found in the group who declared increased alcohol consumption during the pandemic (5.1% and 26%, respectively), with 70% of this group also presenting some form of insomnia symptoms (subthreshold, moderate and severe, combined). On the other hand, the lowest general prevalence of insomnia symptoms (subthreshold, moderate and severe, combined) was observed in the group who never used alcohol (51%). The results are described in detail in Table 5.

Tobacco|The majority of the respondents (63%, *n* = 703) reported that they have never smoked at all, while 16% (*n* = 179) reported a decrease in tobacco consumption during the pandemic, 11% (*n* = 120) reported an increase, and 10% (*n* = 109) declared that they had made no change in smoking during the pandemic. The highest percentage of severe insomnia symptoms (5.8%) was found in the group that reported an increase in tobacco consumption during the pandemic. In turn, the lowest general prevalence of insomnia symptoms (subthreshold, moderate and severe, combined) was found in those who reported that they have never smoked (54%). The post hoc analysis revealed significant differences between the groups reporting an increase in tobacco consumption as well as a decrease and the group that never smoked, but not between the other groups. The results are described in detail in Table 5.

Cannabinoids|The majority of the respondents (73%, *n* = 803) reported that they have never used cannabinoids at all, while 15% (*n* = 175) reported a decrease in cannabis use during the pandemic, 7% (*n* = 79) reported they had made no change in the frequency of cannabis use, and 5% (*n* = 54) reported an increase in frequency compared with the prepandemic period. The post hoc analysis revealed significant differences between the group that reported increased cannabinoids consumption and those who never used cannabinoids at all. Between these groups, the highest prevalence of severe and moderate insomnia symptoms was found in those who reported increased cannabis use (9.3% and 30%, respectively). This group also experienced some form of sleep disturbance (subthreshold, moderate and severe, combined), with only 30% reporting no sleep problems, compared with the 43% respondents of the group who reported no cannabinoid use at all. The results are described in detail in Table 5.

Tranquilizers|The majority of the respondents (75%, *n* = 833) reported that they never used tranquilizers, while 12% (*n* = 130) reported an increase in the frequency of tranquilizer use during the pandemic, 8% (*n* = 92) reported no change in the frequency of use compared to the prepandemic period, and 5% (*n* = 56) declared that they had used tranquilizers less often than before the pandemic. The highest severe and moderate insomnia symptoms were found in the group who reported increased tranquilizer use compared with the prepandemic period (11% and 44%, respectively), and 85% of the respondents of this group reported some form of sleep disturbance. The lowest prevalence of any symptoms of insomnia (51%) was found in the group of respondents who declared that they had never taken tranquilizers at all. The results are described in detail in Table 5.

#### 3.1.4. Relationship between Sleeping Patterns, Physical Activity, and Insomnia Symptoms Severity

**Sleeping patterns|**The majority of the studied group (*n* = 703, 63%) declared that they sleep more compared with the prepandemic period, while 21% (*n* = 234) declared that they noticed no change in the duration of sleeping during the pandemic, and 16% (*n* = 174) declared that they sleep less. The group who declared having less sleep had the highest prevalence of severe and moderate insomnia symptoms (14% and 39%, respectively). The lowest insomnia symptoms scores were found in the group who made no change in the amount of sleep (51%, absence of symptoms). The post hoc analysis revealed significant differences between those who slept less and those who had made no change in sleep duration, as well as those who increased their sleeping time. Similarly, significant differences were found between those who made no change in sleeping time and those who increased the amount of sleep. The results are described in detail in Table 6.

**Physical activity|**The majority of the studied group (*n* = 661, 59%) reported a decrease in physical activity compared with the prepandemic period, while 27% (*n* = 300) reported that they had increased their physical activity, and 14% (*n* = 150) reported that they had made no change. The highest insomnia symptoms were found in the group who reported a decrease in physical activity during the pandemic (subthreshold, moderate and severe), whereas the lowest prevalence of insomnia symptoms was found in the group who reported an increased level of physical activity (absence of insomnia symptoms in 52%). Interestingly, the post hoc analysis revealed significant differences between those who reported a decrease in physical activity during the pandemic and those who made no change in the level of physical activity, as well as between those who increased and decreased their physical activity, but not between those who increased their physical activity and those who made no change. The results are described in detail in Table 6.

## 4. Discussion

### 4.1. Main Findings and Context of the Research

The results of this study confirm, as expected, the high prevalence of insomnia, PTSD, psychopathological symptoms, and psychological distress in the studied population of university students. These are in line with the findings of previous studies reporting a high prevalence of insomnia, anxiety, and depressive symptoms in the population, among other adverse mental health outcomes during the COVID-19 pandemic [2,23,28,29,30,31,32].

Studies that were conducted on insomnia during the COVID-19 pandemic, however, have rarely investigated the origin of sleep problems and have reported them as resulting from the pandemic crisis. This study aimed to address several variables related to poorer sleep quality and establish how they have changed during the pandemic as well as how those changes affected sleeping problems in the studied sample during that time.

At first, certain limitations and intercorrelations between the measures have to be addressed. Apart from ISI, both of the remaining measures used in this study encompass items referring to sleep difficulties. GHQ-28 has a separate subscale addressing “anxiety and insomnia” containing two items related to sleep: “losing much sleep over worry” and “having difficulty in staying asleep once you fall asleep”. The IES-R contains three sleep-related items: “I had trouble staying asleep”, “I had trouble falling asleep, and” I had dreams about it [the event], or something similar. The observed positive correlations in this study may be partially influenced by the aforementioned similarities in the measurements; these, however, cannot be removed without harm to the integrity of the psychometric properties of the questionnaires. Another point of discussion lies with the ability of ISI to detect and discriminate between different forms of sleep disorders. Although the questionnaire is helpful for epidemiological purposes to detect insomnia symptoms and evaluate their severity, it is not able to differentiate between primary and comorbid insomnia [26], nor can it be treated as a diagnosis. This is especially important due to the large battery of literature pointing to the pandemic’s influence on specific sleep disorders, such as obstructive sleep apnea [33] and circadian changes [34]. The ISI scores described in this study should therefore be treated as a reflection of the respondents’ subjective sleep difficulties and their severity, not as a diagnosis of a specific sleep disorder. Apart from a high prevalence of insomnia symptoms, the results of this study also demonstrated that the majority of the studied samples, in fact, declared having slept more during the pandemic than before. This result is in line with the findings of Alfonsi et al. (2021), who also observed an increase in sleep duration during the pandemic, also coupled with better daytime functioning [35]. The aforementioned result further underlines the importance of an accurate assessment of different sleep domains as the associations between the pandemic and sleep quality seem to be more complex than just the presence of insomnia symptoms or a lack thereof.

Another point of discussion is related to the characteristics of the studied population. It has been shown that university students are at an increased risk of experiencing insomnia and sleep difficulties [15,36] as well as psychological distress and mental health problems [37] even before the pandemic. In the present study, 58% of students reported some form of insomnia (subthreshold, moderate and severe, combined), which is in line with the results of the study by Becker et al., conducted in 2010 who investigated sleep problems in a large sample of multiuniversity college students, with 62% of the respondents reporting poor sleep [36]. In another study, where data collection took place before the pandemic, approximately 30% of the studied sample of college students met the clinical cutoff for insomnia [38]. Interestingly, such a high prevalence of sleeping problems was also noted in both of the aforementioned studies [36,38] in 2018, i.e., before the pandemic, which confirms that this population is particularly vulnerable to sleeping problems regardless of the environmental circumstances. Some explanations for the higher prevalence of insomnia and sleep problems among university students include poor sleep hygiene as well as a biologically different circadian preference (chronotype) for later sleep and waking times that may interfere with their daily schedule resulting in even less nighttime sleep [39]. In addition to their high risk for developing mental and sleep disorders, university students also follow a different lifestyle due to their age and college experience. Apart from working part-time and attending classes, this is a time at which social life is at the center of daily schedules, in terms of not only meeting people after class hours, as it typically is later in life, but also living with people in dorms, corented flats, during classes, and semester breaks. This aspect of university life was extremely affected by the pandemic-related lockdowns and social distancing norms. The solitude imposed by the pandemic would have caused an immense change in this population in comparison with other social groups. Previous studies exploring the impact of loneliness on insomnia have demonstrated an interrelationship between the two variables [40], and later studies conducted during the COVID-19 pandemic also confirmed this association [41]. However, it remains unclear how loneliness affects insomnia. Some explanations point to increased physiological arousal and activation of the hypothalamic-pituitary-adrenal axis as a result of perceived loneliness, leading to a negative impact on the ability to sleep. The bidirectionality of the relationship has been explained by excessive concerns about isolation during extended waking times in people with insomnia, as well as the impairment of social activities by sleep deprivation [40].

### 4.2. The Relationships between PTSD Symptoms and Insomnia Symptoms

The relationship between PTSD and insomnia has been widely investigated by various researchers [42,43,44,45,46]. Although insomnia and PTSD are two distinct conditions, the inclusion of insomnia in the diagnostic criteria of PTSD, as well as a high comorbidity rate between PTSD and insomnia of 30–60% [45], indicates some overlap between the two disorders. A study on combat veterans revealed that individuals with insomnia symptoms are at a greater risk of developing PTSD [47] following a traumatizing event, defining insomnia as a preceding factor, as well as indicating that insomnia symptoms may occur if individuals experience a traumatic event, with or without the development of full-scale PTSD [48]. According to the hyperarousal model of insomnia [49], a combination of physiological and psychological factors plays a role in the development of insomnia. The model assumes that insomnia symptoms may occur as a result of a negative event. However, the chronic nature of disordered sleep is maintained by both classical conditioning in relation to the negative associations between sleep and maladaptive behaviors affecting sleep (substance use and changes in bedtime), and conditioned arousal (cognitive, somatic, and cortical activation), or through an increased autonomic activity that may lead to sleep difficulties [49,50,51]. The results of the present study suggest not only that PTSD symptoms were related to insomnia symptoms but also that insomnia symptoms increased with an increase in PTSD symptoms. Interestingly, this correlation was statistically more significant for the intrusion and arousal subscales of the IES-R. The intrusion subscale includes items concerning flashbacks, dreams, sleep, and thoughts about the traumatizing event, while the hyperarousal subscale contains items relating to emotions (irritability, fear), somatic symptoms, alertness, and difficulty concentrating. As mentioned above, elevated insomnia scores could be interpreted as an outcome of a traumatic experience (in this case, the changes induced by the pandemic in the environment) in itself, as a symptom of developing PTSD, or both. In the third scenario, insomnia symptoms would have been present in the individuals even before the pandemic, precipitating the development of negative stress responses (i.e., PTSD symptoms) and possibly aggravating those responses but presenting as a disorder on its own.

Another result that supports the assumption of a partially separate origin of the insomnia symptoms found in this study is the discrepancies in the relationship between psychological distress, PTSD, and insomnia symptoms severity. However, the findings of the study confirmed the already well-established relationship between the occurrence of psychopathological symptoms and insomnia [52,53,54,55,56]. The study showed that the majority of respondents who reported no insomnia (absence of symptoms) also scored above the cutoff for psychological distress. Similarly, the second largest group who did not present any insomnia symptoms were those who also experienced severe PTSD symptoms. A few possible explanations can be given for these findings. Firstly, since the study did not control for sleep medication, some respondents of the group who experienced PTSD symptoms and other psychopathological symptoms might have already sought help for their mental health conditions and been taking sleep medications, which in turn could have led to false negative responses for the questions on the subjective experience of insomnia. However, the respondents were asked about the use of tranquilizers, and the highest insomnia symptoms were noted in the group who reported increased use of tranquilizers during the pandemic. This indicates that those substances were insufficient to improve sleep in a manner that would make the respondents change their answers on the insomnia scale, which renders this explanation unlikely. Secondly, significant differences were observed between the number of participants in each of the studied groups. Nearly 80% of the entire studied sample scored above the cutoff for psychological distress, and only 20% scored below the cutoff at the time of data analysis. The same trend was noted for the PTSD symptoms, with nearly 50% of the studied group experiencing severe PTSD symptoms, and only 26% reported no PTSD symptoms at all. The group that reported no insomnia symptoms was larger but was still less than half of the studied population (42%). Although these results clearly indicate the impact of the pandemic on students’ mental health, they also suggest that the relationship between no insomnia symptoms, no PTSD symptoms, and no psychological distress has to be weak within the studied population. If the results are assumed to be accurate despite the previously mentioned methodological points, it can be concluded that insomnia in the studied group could be an entirely separate disorder, which is unrelated to PTSD and psychopathological symptoms, and even the pandemic. This conclusion is partially supported by another finding that 49% of the studied population who made no change to their sleeping patterns during the pandemic declared some form of insomnia symptoms (subthreshold, moderate, or severe), and that moderate and severe insomnia symptoms seemed to be associated with a further decrease of time spent sleeping, rather than a change by itself.

The associations with pandemic-related dreams and psychological distress (GHQ), as well as insomnia symptom severity, confirm the findings on oneiric activity during the pandemic. Although the direction of the relationship cannot be determined, the study by Gorgoni et al. (2021) points to an increased dream frequency during the pandemic, with higher dream frequencies noted, among others, for subjects with poor sleep quality and depressive symptoms [57]. Similarly, Scarpelli et al. (2021) found sleeping problems, anxiety, and depression to be some of the predictors of higher nightmare frequency [58]. Another longitudinal study by Scarpelli (2021) using dream diaries demonstrated an increased dream recall frequency as well as the frequency of lucid dreams and pandemic-related dream content [59]. The authors point to those findings in association with the traumatic context of the pandemic as being associated with a “collective trauma” of this stressful period.

### 4.3. The Relationships between Substance Use, Physical Activity, and Insomnia Symptoms

The association between alcohol use and sleep disturbance is commonly known. Among others, alcohol interferes with REM sleep and contributes to short sleep duration, breathing-related sleep disorders, disruption of circadian rhythms, and the onset of insomnia [60]. The relationship between alcohol use and insomnia is also bidirectional, with alcohol contributing to sleep disturbances and insomnia at baseline being associated with heavy drinking [61]. In this study, 70% of the group who reported increased alcohol consumption due to the pandemic exhibited some form of insomnia symptoms. Interestingly, the majority of the respondents (43%) reported that they had decreased alcohol consumption compared with the prepandemic period. In the previous study, this result was explained by environmental changes, as lockdowns in Poland at the time of the study prevented much of the social activities that were associated with alcohol consumption among university students [23]. Assuming that alcohol and insomnia have an influence on each other, it is unclear whether sleeping problems were a result of alcohol consumption or whether alcohol was used as a self-medication for insomnia. However, it can be understood that the increase in alcohol consumption was deliberate as spontaneous drinking occasions were almost entirely off-limits at the time of the study. Although cannabis is commonly used as a (self-)medication for insomnia, as well as for other ailments, research on the effect of cannabis on sleep has yielded mixed and often contradictory results. Reviews on cannabis use and insomnia unanimously suggest that cannabis has an impact on sleep, although the exact picture of the beneficial and detrimental effects of cannabis is largely inconclusive [62,63]. The results of the present study suggested a link between an increase in cannabis use during the pandemic and the presence of moderate-to-severe insomnia symptoms. On the one hand, an increase in cannabis use and insomnia can be interpreted as indicating the possible detrimental effects of cannabis on sleep. The second explanation for this result comes from the study by Babson et al., who noted that the risk of relapse of insomnia symptoms is greater in people who previously reduced their cannabis use [64,65]. This indicates that an increase in cannabis use could be caused by insomnia symptoms rather than the other way around.

Regarding physical activity, the highest insomnia scores in this study were found in the group who reported a decrease in physical activity, whereas the lowest prevalence of insomnia was found in the group who reported an increase in the amount of physical activity. This finding is in line with the results of the metanalysis by Kredlow et al., which showed that physical exercise has a significant beneficial effect on overall sleep quality [66]. In the studied sample, significant differences were found between those who decreased their physical activity compared with those who made no change as well as between those who increased and those who decreased their physical activity, but not between those who increased and those who made no change. In line with other studies, it can be hypothesized that the group which made no change may consist of people who already have a steady routine of physical exercise and therefore benefit from the effects of regular physical activity, which, according to Kredlow et al., has an even more beneficial subjective and objective effect on sleep, comparable to behavioral therapy or pharmacotherapy for insomnia [66].

## 5. Limitations

The major limitation of this study is the epidemiological situation at the time of data collection. Although data for the study were collected using the CAWI methodology, some limitations related to the online format of the questionnaire remain. An older demographic may be underrepresented, and there may be a selection bias in terms of institutions willing to make the study accessible to their students. The latter was partially controlled by distributing the survey link among student organizations and social media to avoid relying solely on the university media. Another limitation refers to the sample’s representativeness—the data were collected through internet-based distribution, and participation was determined by the willingness to complete the questionnaire or lack thereof. It is not possible to determine whether this is a representative sample of Polish university students as all of the collected data came from students who volunteered to take part in the survey, and the reasons for participation or refusal to participate were not obtained. The generalizability of the results may also be influenced by the gender imbalance found in the study in favor of the female gender. Although there were no statistically significant differences between the studied variables in terms of gender, the results may be subjected to the influence of a largely female sample. The collected data did not allow exploring the direction of the relationships between the studied variables. Information on the direction of influence would significantly improve the understanding of the relationship, and therefore more studies are needed on this in the future. Due to the anonymity of data collection, no follow-up data can be obtained, and no control group was recruited. As there was no control group, the results should be interpreted with caution. The study is based only on subjective self-reports, as no objective data were collected, so the results should be interpreted as a reflection of the participants’ self-experiences. On the other hand, subjective assessment is the only method of analysis that allows investigating the severity of symptoms from the standpoint of the affected person or the person experiencing discomfort. Another limitation concerns the instruments used in the study and their interpretation as well as discriminative value. Both the ISI and IES-R are not designed to diagnose insomnia or PTSD, nor to differentiate between different sleep disorders that may occur in the studied population. Both instruments are merely a reflection of the subjective experience of the presence of symptoms of PTSD or sleeping difficulties. The presented ISI and IES-R scores should therefore not be treated as diagnoses but subjective complaints related to the presence of sleeping difficulties and PTSD-like symptoms.

## 6. Conclusions

The majority of the findings of this study are in line with recent studies on the pandemic, as well as studies on factors associated with poor sleep quality in general. The findings point to a high prevalence of psychopathological symptoms (e.g., depression, anxiety, PTSD symptoms) as well as insomnia symptoms during that time and relationships between substance use, physical activity, and insomnia symptoms. Apart from confirming previous findings, we were able to establish the pandemic’s influence on some of the variables, i.e., an increase of substance consumption or decrease of physical activity, as well as the presence of pandemic-related dreams on insomnia symptoms and a general increase in sleep time specifically related to the pandemic. As the study design did not allow for directional assumptions, most of the presented variables can be understood in the context of both cause and effect in relation to the established insomnia symptoms. The limitations of the study design simultaneously underline the need for further research that would include an accurate assessment of insomnia symptoms as well as allow for conclusions on directionality, as the intuitive assumption that insomnia is an outcome of the pandemic seems to be insufficient in understanding the relationship.

## Figures and Tables

**Table 1:** Demographic characteristics and distribution of insomnia symptoms severity (Insomnia Severity Index, ISI) in the study sample *n* = 1111.

Characteristic	*n*	% of *n*	Insomnia Symptoms Severity (ISI)			
Demographic			Absence, *n* = 469 ^1^	Sub-Treshold, *n* = 402 ^1^	Moderate, *n* = 199 ^1^	Severe, *n* = 41 ^1^
**Gender**						
**Male**	269	24.21%	129.00 (27.51%)	92.00 (22.89%)	40.00 (20.10%)	8.00 (19.51%)
**Female**	842	75.79%	340.00 (72.49%)	310.00 (77.11%)	159.00 (79.90%)	33.00 (80.49%)
**Age**	Mean 22.20, SD 2.42, median 22.00 (21.00, 23.00)		22.31 (2.22), 22.00 (21.00, 23.00)	22.14 (2.47), 22.00 (20.00, 23.00)	22.16 (2.83), 22.00 (20.00, 23.00)	21.93 (2.07), 22.00 (20.00, 23.00)
**Place of residence**						
**<100 thousand**	531	47.79%	213.00 (45.42%)	198.00 (49.25%)	102.00 (51.26%)	18.00 (43.90%)
**>100 thousand**	580	52.21%	256.00 (54.58%)	204.00 (50.75%)	97.00 (48.74%)	23.00 (56.10%)
**Employment**						
**Unemployed**	832	74.89%	356.00 (75.91%)	301.00 (74.88%)	145.00 (72.86%)	30.00 (73.17%)
**Employed**	279	25.11%	113.00 (24.09%)	101.00 (25.12%)	54.00 (27.14%)	11.00 (26.83%)
**Source of income**						
**Partner**	27	2.43%	13.00 (2.77%)	7.00 (1.74%)	5.00 (2.51%)	2.00 (4.88%)
**Family**	929	83.62%	396.00 (84.43%)	342.00 (85.07%)	158.00 (79.40%)	33.00 (80.49%)
**Self-supportive**	155	13.95%	60.00 (12.79%)	53.00 (13.18%)	36.00 (18.09%)	6.00 (14.63%)
**Relationship**						
**Single**	528	47.52%	222.00 (47.33%)	185.00 (46.02%)	109.00 (54.77%)	12.00 (29.27%)
**Married**	25	2.25%	12.00 (2.56%)	8.00 (1.99%)	4.00 (2.01%)	1.00 (2.44%)
**In** **a relationship**	558	50.23%	235.00 (50.11%)	209.00 (51.99%)	86.00 (43.22%)	28.00 (68.29%)

^1^ *n* (%); Mean (SD) Median (IQR).

**Table 2:** Relationship between psychological distress General Health Questionnaire-28 (GHQ-28) and insomnia severity (Insomnia Severity Index, ISI), *n* = 1111.

			GHQ Somatic Symptoms		GHQ Anxiety and Insomnia		GHQ Social Dysfunction		GHQ Severe Depression		GHQ Total	
			Mean (SD)	Effect Size (η^2^), *p* ^1^	Mean (SD)	Effect Size (η^2^), *p* ^1^	Mean (SD)	Effect Size (η^2^), *p* ^1^	Mean (SD)	Effect Size (η^2^), *p* ^1^	Mean (SD)	Effect Size (η^2^), *p* ^1^
**ISI**	1	Absence	6.06 (3.64)	0.30514 *	8.33 (4.83)	0.27759 *	10.09 (4.29)	0.11136 *	4.87 (5.01)	0.16575 *	29.35 (14.78)	0.27227 *
	2	Subthreshold	9.58 (4.05)		12.57 (4.68)		12.54 (4.16)		8.53 (5.61)		43.23 (15.22)	
	3	Moderate	12.20 (3.91)		15.08 (3.90)		13.80 (4.34)		10.43 (5.79)		51.51 (14.75)	
	4	Severe	14.78 (4.20)		17.27 (3.83)		13.90 (4.04)		10.76 (6.36)		56.71 (14.41)	
**Post hoc** **analysis**	Statistically significant, except		*-*		*-*		2.4; 3.4				-	

^1^*p*-value: * ≤ 0.001.

**Table 3:** Relationship between PTSD symptoms (Impact of Events scale-Revised, IES-R, and subscales) and insomnia severity (Insomnia Severity Index, ISI), *n* = 1111.

			IES-R Intrusion		IES-R Avoidance		IES-R Hyperarousal		IES-R Total	
			Mean (SD)	Effect Size (η^2^), *p* ^1^	Mean (SD)	Effect Size (η^2^), *p* ^1^	Mean (SD)	Effect Size (η^2^), *p* ^1^	Mean (SD)	Effect Size (η^2^), *p* ^1^
**ISI**	1	Absence	9.55 (7.27)	0.16, *	10.95 (7.10)	0.04, *	7.02 (4.97)	0.24, *	27.61 (17.24)	0.16, *
	2	Subthreshold	14.53 (7.33)		13.23 (6.45)		11.37 (5.42)		39.12 (16.72)	
	3	Moderate	17.69 (7.75)		14.57 (6.82)		14.28 (5.21)		46.64 (16.93)	
	4	Severe	19.18 (8.10)		14.55 (6.98)		16.45 (5.60)		50.18 (18.74)	
**Post hoc analysis**	Statistically significant, except		3.4		2.3; 2.4; 3.4		-		3.4	

^1^*p*-value: * ≤ 0.001.

**Table 4:** Relationship between psychological distress (General Health Questionnaire-28, GHQ-28 score > 24 points), PTSD severity (Impact of Events scale-Revised, IES-R), and insomnia severity (Insomnia Severity Index, ISI), *n* = 1111.

			ISI						
			Absence	Subthreshold	Moderate	Severe	Total	Chi-Squared Test, *p* ^1^	
			*n* (%)	*n* (%)	*n* (%)	*n* (%)	*n* (%)		
**GHQ-28**	1	<24	190 (40.5%)	47 (11.7%)	5 (2.5%)	1 (2.4%)	243 (21.9%)	172.4453 *	
	2	≥24	279 (59.5%)	355 (88.3%)	194 (97.5%)	40 (97.6%)	868 (78.1%)		
**IES-R**	1	No PTSD	187 (39.9%)	77 (19.2%)	18 (9.0%)	3 (7.3%)	285 (25.7%)	29.83978 *	
	2	Probable PTSD	36 (7.7%)	25 (6.2%)	14 (7.0%)	4 (9.8%)	79 (7.1%)		
	3	Subclinical PTSD	70 (14.9%)	57 (14.2%)	14 (7.0%)	2 (4.9%)	143 (12.9%)		
	4	Severe PTSD	147 (31.3%)	228 (56.7%)	142 (71.4%)	29 (70.7%)	546 (49.1%)		
	5	Unknown	29 (6.2%)	15 (3.7%)	11 (5.5%)	3 (7.3%)	58 (5.2%)		

^1^*p*-value: * ≤0.001.

**Table 5:** Relationship between insomnia severity (Insomnia Severity Index, ISI) and substance use, *n* = 1111.

			ISI						
			Absence	Subthreshold	Moderate	Severe	Total	Chi-Squared Test, *p* ^1^	
			*n* (% of *n*)	*n* (%)	*n* (%)	*n* (%)	*n*		
**Alcohol consumption**	1	More often than before the Pandemic	59 (30%)	77 (39%)	52 (26%)	10 (5.1%)	198	31.05, **	
	2	No change	138 (47%)	114 (39%)	37 (12%)	7 (2.4%)	296		
	3	Less often than before the Pandemic	204 (43%)	175 (37%)	81 (17%)	19 (4%)	479		
	4	I have never used	68 (49%)	36 (26%)	29 (21%)	5 (3.6%)	138		
**Use of tobacco**	1	More often than before the Pandemic	26 (22%)	54 (45%)	33 (28%)	7 (5.8%)	120	32.77, **	
	2	No change	38 (35%)	51 (47%)	17 (16%)	3 (2.8%)	109		
	3	Less often than before the Pandemic	80 (45%)	61 (34%)	31 (17%)	7 (3.9%)	179		
	4	I have never used	325 (46%)	236 (34%)	118 (17%)	24 (3.4%)	703		
**Use of marijuana**	1	More often than before the Pandemic	16 (30%)	17 (31%)	16 (30%)	5 (9.3%)	54	22.49, *	
	2	No change	28 (35%)	34 (43%)	17 (22%)	0 (0.0%)	79		
	3	Less often than before the Pandemic	78 (45%)	55 (31%)	31 (18%)	11 (6.3%)	175		
	4	I have never used	347 (43%)	296 (37%)	135 (17%)	25 (3.1%)	803		
**Use of tranquilizers**	1	More often than before the Pandemic	19 (15%)	40 (31%)	57 (44%)	14 (11%)	130	127.31, **	
	2	No change	23 (25%)	43 (47%)	19 (21%)	7 (7.6%)	92		
	3	Less often than before the Pandemic	22 (39%)	22 (39%)	11 (20%)	1 (1.8%)	56		
	4	I have never used	405 (49%)	297 (36%)	112 (13%)	19 (2.3%)	833		

^1^*p*-value: * ≤0.01, ** ≤0.001.

**Table 6:** Relationship between sleeping patterns, physical activity, and insomnia severity (Insomnia Severity Index, ISI), *n* = 1111.

			ISI						
			Absence	Subthreshold	Moderate	Severe	Total	Chi-Squared Test, *p* ^1^	
			*n* (% of *n*)	*n* (% of *n*)	*n* (% of *n*)	*n* (% of *n*)	*n*		
**Sleep**	1	sleep more	321 (46%)	269 (38%)	99 (14%)	14 (2%)	703	144.62, *	
	2	No change	119 (51%)	80 (34%)	32 (14%)	3 (1.3%)	234		
	3	sleep less	29 (17%)	53 (30%)	68 (39%)	24 (14%)	174		
**Physical activity**	1	Increased level	157 (33.5%)	92 (22.9%)	47 (23.6%)	4 (9.8%)	300	29.84, *	
	2	No change	72 (48%)	52 (35%)	23 (15%)	3 (2%)	150		
	3	Decreased level	240 (36%)	258 (39%)	129 (20%)	34 (5.1%)	661		

^1^*p*-value: * ≤0.001.

## Data Availability

Datasets are available on request. The raw data supporting the conclusions of this article will be made available by the authors. Further inquiries can be directed to the corresponding author.
